# NCMW: A Python Package to Analyze Metabolic Interactions in the Nasal Microbiome

**DOI:** 10.3389/fbinf.2022.827024

**Published:** 2022-02-25

**Authors:** Manuel Glöckler, Andreas Dräger, Reihaneh Mostolizadeh

**Affiliations:** ^1^ Department of Computer Science, University of Tübingen, Tübingen, Germany; ^2^ Computational Systems Biology of Infections and Antimicrobial-Resistant Pathogens, Institute for Bioinformatics and Medical Informatics (IBMI), University of Tübingen, Tübingen, Germany; ^3^ German Center for Infection Research (DZIF), Partner Site Tübingen, Tübingen, Germany; ^4^ Cluster of Excellence “Controlling Microbes to Fight Infections”, University of Tübingen, Tübingen, Germany

**Keywords:** microbial communities, nasal microbiome, computational biology, genome-scale modeling, constraint-based modeling

## Abstract

The human upper respiratory tract is the reservoir of a diverse community of commensals and potential pathogens (pathobionts), including *Streptococcus pneumoniae* (pneumococcus), *Haemophilus influenzae*, *Moraxella catarrhalis*, and *Staphylococcus aureus*, which occasionally turn into pathogens causing infectious diseases, while the contribution of many nasal microorganisms to human health remains undiscovered. To better understand the composition of the nasal microbiome community, we create a workflow of the community model, which mimics the human nasal environment. To address this challenge, constraint-based reconstruction of biochemically accurate genome-scale metabolic models (GEMs) networks of microorganisms is mandatory. Our workflow applies constraint-based modeling (CBM), simulates the metabolism between species in a given microbiome, and facilitates generating novel hypotheses on microbial interactions. Utilizing this workflow, we hope to gain a better understanding of interactions from the metabolic modeling perspective. This article presents nasal community modeling workflow (NCMW)—a python package based on GEMs of species as a starting point for understanding the composition of the nasal microbiome community. The package is constructed as a step-by-step mathematical framework for metabolic modeling and analysis of the nasal microbial community. Using constraint-based models reduces the need for culturing species *in vitro*, a process that is not convenient in the environment of human noses.

**Availability:** NCMW is freely available on the Python Package Index (PIP) *via* pip install NCMW. The source code, documentation, and usage examples (Jupyter Notebook and example files) are available at https://github.com/manuelgloeckler/ncmw.

## 1 Introduction

The human nose community is far more than a convenient model system, while it plays vital roles in human health and global nutrient cycles (Widder et al., 2016). The previous work focusing on ecological interactions can identify whether the interacting partners promote or hinder each other’s growth (Carrara et al., 2015). Studying these interactions on their own does not reveal the mechanistic details of the nasal microbiome community or its composition and structure. The interaction mechanisms, either potential competition or cooperation of species in the community, can determine the species assembly processes in the community. Understanding the pair-wise or multispecies interactions in the human nose community is a starting point for manipulating microbiomes for therapeutic and prophylactic purposes. However, this is often made difficult due to the inability to culture and co-culture the various species of human nasal microbiota *in vitro*.

The study on human gut microorganisms and their community has received much attention compared to that on other communities. Different approaches such as high-throughput studies, modeling methodologies such as GEMs (Klitgord and Segrè, 2010; Freilich et al., 2011; Zomorrodi and Maranas, 2012; Seif et al., 2019; Diener et al., 2020), AGORA (Levy and Borenstein, 2013), community and systems-level interactive optimization (CASINO) (Levy and Borenstein, 2014), BacArena (Bauer et al., 2017), and GutLogo (Lin et al., 2018), and databases such as virtual metabolic human database (VMH) (Noronha et al., 2019), have been developed on microbial communities mainly focusing on the four human sites, such as gut, oral, skin, and vaginal. Although resources on human nose research have been limited, a direct application of existing modeling pipelines available for the gut is consequently difficult due to both aerobic and anaerobic living conditions for human nose bacteria (Man et al., 2017; Proctor and Relman, 2017). Some researches represent the role of nasal microbial inhabitants in inhibiting the pathogens or turning commensal into a pathogen (Yan et al., 2013; Liu et al., 2015; Bomar et al., 2018). However, factors that influence pathogens have not been defined yet. A synthetic nasal medium (SNM3) has been developed by Krismer et al. (2014) that permits consistent growth of *S. aureus* isolates. Yet, there are still members of the nasal microbiome that remain unculturable even in this medium. Using this medium simulates the bacterial growth within the human nose. In addition, GEMs and constraint-based modeling are used to biochemically and physicochemically predict the phenotype from genotype without the need for a bacterial culture of species in the lab (Shoaie et al., 2013; Cook and Nielsen, 2017; van der Ark et al., 2017).

Here, we developed a workflow to construct a predictive computational model of the human nose microbial community and its interactions. The workflow is highly flexible and can adapt to changes or updates whenever yet unexplored or modified models are added. This can be used for pair-wise interactions and a more extensive system with more species. However, the computational complexity increases with the number of species. In particular, this is the first time a comprehensive flux balance analysis workflow has been created for the nasal human microbial community. The workflow relies on a multilevel and multi-objective optimization problem to grasp commensal, competitive, or further interactions. Like OptCom (Zomorrodi and Maranas, 2012), a multilevel optimization framework for the metabolic modeling, we also considered species-level and community-level criteria in defining the objective function. Therefore, the optimal growth of each species in the community and the optimal growth of the entire community have been taken into account. Additionally, we explicitly included different weights in the definition of the objective function of the community based on the growth ratio of species in the community to estimate more realistic and quantitative metabolic predictions.

In NCMW, we integrate diverse types of available definitions for media those are subsets of the leading preferred media, e.g., SNM3. They may simulate environmental conditions that favor commensal or competitive interactions (Carrara et al., 2015). These different media definitions facilitate knowledge interchange within the theoretical, experimental, and computational definition of the human nose environment. This example demonstrates that systematic model-based analysis has the ability to detect such potential nutrients in a much more efficient way in adoption between wet and dry labs. In addition, adding any newly defined media to our workflow is feasible. Hence, the aforementioned one represents the compatibility of our package for different communities such as the gastrointestinal tract, skin, and vagina, in the case of the definition of respected media. This strategy is entirely implemented as a Python package and available as an open-source software package named NCMW. It facilitates integrating genome-scale metabolic models in the human nose environment for understanding the complexity of this community.

To validate our work and demonstrate the ability of NCMW, it was applied on the nasal microbial community involving *Staphylococcus aureus* and *Dolosigranulum pigrum* (Mostolizadeh et al., 2022). Next, NCMW is also employed to model the more complex system of the five abundant nasal microbial to show different applications of the package on a large community and documented as an example to the package.

Researchers can easily incorporate, access, modify, and extend our tool to develop their research protocols for scientific analysis on the community. The workflow discussed here lays the foundation for future testing of human nasal species as potential probiotics to prevent or antagonize colonization of the nares by pathogens, especially *Staphylococcus aureus*. We believe that NCMW can unravel some biological questions on how the nasal community shapes and the members can have widely different effects on each other.

## 2 Materials and Methods

The workflow’s essential methods and approaches depend on computational models for inferring fluxes in biochemical networks. In order to estimate the metabolic fluxes of the model organism, we use GEMs. The approach used here is therefore based on high-quality genome-scale models generated for species within the community, which we will discuss in more detail later.

### 2.1 Flux Balance Analysis and Flux Variability Analysis

Flux balance analysis (FBA) is a linear optimization method that maximizes a biologically motivated objective function. Biomass production, for instance, is a widely used objective that facilitates calculations of an organism’s growth rate. Other examples include the production rate of a biotechnologically relevant metabolite. A meaningful objective function enables flux balance analysis (FBA) to predict the flow of matter through the network (Orth et al., 2010).

Flux variability analysis (FVA) calculates the minimal and maximal possible flux through a reaction channel in the model. Simultaneously, flux variability analysis (FVA) maintains a specific network state. For instance, it might sustain 90% of the maximal possible biomass production rate. Such analysis is particularly useful for analyzing the robustness of metabolic models (Gudmundsson and Thiele, 2010).

### 2.2 Community Approaches

There are different approaches for creating a community model. They are categorized as compartmentalized, pooled reactions, and nested analysis methods.

#### 2.2.1 Pooled Community

This approach combines metabolic reactions and metabolites from all members of a species in one shared compartment. In cases where more than one species catalyzes the reaction, only one reaction is assigned. Although this community minimizes the computational burden, it has the weakness of not specifying which species use or produce a particular enzyme or metabolite (Taffs et al., 2009). As a result, the model can be used to investigate the community’s growth but not to analyze the interaction between community members.

#### 2.2.2 Shuttle Community

In this approach, each exists in a separate compartment. Individual species then interact in an additional “external” compartment, representing the extracellular space. This shared external space allows for interspecies interaction through “shuttle reactions.” Thus, we can explicitly evaluate how bacterial species will interact with other community members as well as the surrounding environment. Furthermore, the shuttle reactions that identify shared reactions between species can also be defined based on our interests (Klitgord and Segrè, 2010).

#### 2.2.3 Community Optimization

As standard, we interpret community growth as a linear combination of individual’s growth, i.e.,

$$\begin{array}{ll} \max G & =\underset{i}{\sum }w_{i}g_{i}\\ \text{Subject to:}\\ \forall_{i}:S_{i}\nu_{i} & =0\\ S^{\text{sh}+\text{ex}}\nu_{i}^{\text{sh}+\text{ex}} & =0\\ S^{\text{ex}}\nu^{\text{ex}} & =0, \end{array}$$ (1)

where *S*_*i*_, *S* ^sh^ and *S* ^ex^ denote the stoichiometry matrices to ensure mass-balance within any reaction. We denote a vector of reaction fluxes by *ν*.

The internal reactions *ν*_*i*_ are essentially unbounded. We use a lower bound of −1,000 mmol/(gDW⋅h) and an upper bound of 1,000 mmol/(gDW⋅h) as default. The lower bounds for the community exchange reactions *ν* ^ex^ are defined by the medium, e.g., typically −10 mmol/(gDW⋅h). For exchange reactions, we add an associated shuttle reaction that mediates the uptake from the external medium under mass balance constraints, i.e., the sum of individual fluxes must equal the community exchange flux. These reactions allow interaction between community members, i.e., model *i* can produce metabolites for model *j*. We assign each of them a lower bound of −50 mmol/(gDW⋅h) and an upper bound of 1,000 mmol/(gDW⋅h). As default for any metabolite that a model can uptake or secrete, we add associated shuttles. Nevertheless, we also specify the set of reactions for which shuttle reactions should be added (all others are modeled as regular exchanges). This kind of community is also employed in SteadyCom or MICOM (Chan et al., 2017; Diener et al., 2020).

Notice that if *w*_*i*_ *g*_*i*_ = 0, we consider the community member *i* as “dead” and thus would be expected that all associated fluxes are zero, especially all shuttle reactions should have zero flux, as a “dead” community member should not be able to produce or consume metabolites into the external environment. Unfortunately, this behavior is not guaranteed by the constraints imposed. If *w*_*i*_ *g*_*i*_ = 0, any pathway that does not provide biomass *g*_*i*_ can be used to produce metabolites to increase the biomass of other models. This is unrealistic and should be prevented. To do so, we add an additional linear constraint to each shuttle reaction. We impose that $-Cw_{i}g_{i}\leq \nu_{i}^{\text{sh}}\leq Cw_{i}g_{i}$ , whereas *C* is a positive constant which can be interpreted to regularize the effect of this constraint. This clearly ensures that if *w*_*i*_ = 0 or *g*_*i*_ = 0 also $\nu_{i}^{\text{sh}}=0$ . The constraint ensures that the uptakes or secretions of any model must be proportional to their growth, whereas *C* is proportionally constant. If we choose *C* as a large number, i.e., *C* = 10,000, then this constraint has almost no effect if *w*_*i*_ *g*_*i*_ > 0.

Consistently with Diener et al. (Diener et al., 2020), we observed that optimizing this objective can lead to solutions where only a few taxa grew with high growth rates, and all the other community members had growth near zero. Naively, we could enforce all growth *g*_*i*_ ≥ *g* ^min^, yet if a single model is unable to obtain *g* ^min^, then there will be no feasible solution. We alternatively ensure that each community member must reach a certain percentage of the total community growth, i.e., *w*_*i*_ *g*_*i*_ ≥ *αG* with *α* ∈ [0, 1]. Diener et al. (Diener et al., 2020) proposed the cooperative trade-off method, which first maximizes the community objective to obtain *G* ^max^. For a given trade-off *α* ∈ [0, 1], the solution is then obtained by minimizing $\sum_{i}g_{i}^{2}$ under the constraint that *G* ≥ *αG* ^max^. This *ℓ*_2_ regularization promoted a non-sparse solution and was shown to yield more accurate growth estimates for metabolic models in the human gut (Diener et al., 2020).

In addition, by defining different weights in the optimization function for each species, an abundant profile for each species is scaled to predict requirements for inhibiting the pathogens (Chan et al., 2017; Mostolizadeh et al., 2019). This indeed enables the determination of the range of allowable fluxes to impose conditions for increasing or decreasing the commensal or pathogenic species’ growth rate and infers a reasonable presupposition for the experimental test (Chan et al., 2017; Mostolizadeh et al., 2019).

### 2.3 Media

A growth medium is a list of nutrients designed to support the growth of microorganisms. There are different types of media suitable for growing different types of cells. Here, we use a different type of media for growing microbial communities.

#### 2.3.1 The Synthetic Nasal Medium

Krismer et al. (Krismer et al., 2014) developed a unique SNM3, which has been shown to provide all requirements for a variety of different nasal bacteria. SNM3 is an essential step toward an *in vitro* testing system for the human nose and is defined as the default medium in our Python package.

#### 2.3.2 Competition-Inducing Medium and Cooperation-Inducing Medium

First, all exchange metabolites are provided at the minimal required amount computed by FVA. Then, the competition-inducing medium (COMPM) is defined as the ranges of fluxes of the exchange reactions that support its maximal biomass rate (MBR) while they are in the minimal amount (Freilich et al., 2011). Therefore, in this medium, each species is allowed for reaching its maximum biomass rate. However, within a community, any resource overlap should reduce the individual biomass rate.

Although, a cooperation-inducing Medium (COOPM) is defined as a set of metabolites that support only a small positive growth rate such that the removal of any metabolite from the set would force the system to have no such solution (Freilich et al., 2011). These two media are determined as rich and poor media, which are a subset of any main media of interest (based on our default setting, they are a subset of SNM3).

### 2.4 Similarity and Consistency

The nasal microbial community comprises various species living together and plays an essential role in human health. To identify the interaction of members, we would first focus on how the GEMs of species are qualitatively consistent and how similar they are. To detect the consistent part of a network or blocked reactions, an algorithm called FastCC is applied (Vlassis et al., 2014). FastCC is based on standard MatLAB (Vlassis et al., 2014), although this was also adjusted based on a pure LP implementation for cobra users^1^. In addition, the Jaccard coefficient can be used to measure the similarity between the two sets, in comparing the overlap of reactions and metabolites between species (Jaccard, 1912). The Jaccard coefficient can be defined as the size of the intersection of the two sets divided by the size of the union of the two sets (Tan et al., 2016). Therefore, if the Jaccard coefficient equals one, two sets are identical, while zero indicates two disjoint sets.

## 3 Results

### 3.1 Implementation

The core capabilities of NCMW are set up by the installation of the package *via* pip. All dependencies are installed as the Python package NCMW. The only requirement that is not installed (despite obvious ones such as Python) is some solver that can minimize quadratic objectives and is supported by pycobra (Ebrahim et al., 2013) (but also just for some operations).

**FIGURE 1:**
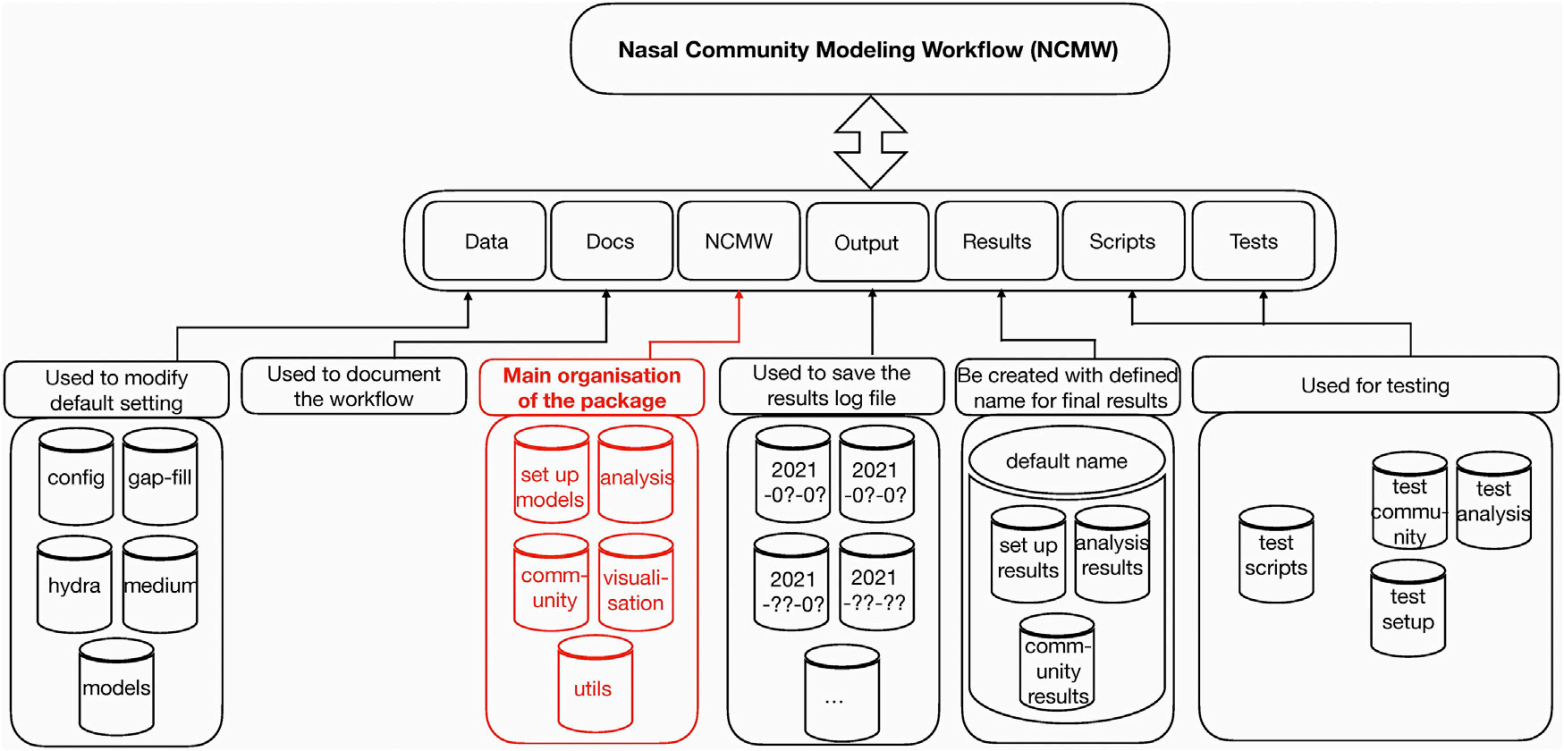
Entire nasal community modeling workflow (NCMW). In the first layer, the package includes seven folders. In the second layer, the tasks which each folder can do are represented. In the last layer, the sub-folders included in each folder are shown. The highlighted part, as the red part, is the main folder of the package. To run the package and get the results, we use command ncmw setup, ncmw analysis, and ncmw community in the command line.

The entire folder NCMW is shown in Figure 1 and is as follows:

NCMW: ▸ Data.

This folder includes four sub folders, namely configs, hydra, medium, and models.

NCMW: ▸ Data ▸ Configs.

These are used for the default setting of maximum lower and upper bound of reactions −1,000 mmol/(gDW⋅h) to 1,000 mmol/(gDW⋅h).

NCMW: ▸ Data ▸ *Hydra*.

We use hydra (Yadan, 2019) to support, e.g., parameter overriding. This folder contains all default parameters for the scripts. All can be overwritten in the console or by modification of the file.

NCMW: ▸ Data ▸ Medium.

This folder contains the default SNM3. Any medium of user interest can be placed here, to the server as a new default medium. All derived media will then be based on the medium specified within the parameters.

NCMW: ▸ Data ▸ Models.

All models of participating species in the community must be accessible in this folder. One can either add additional models to the workflow or create a new folder and properly overwrite the corresponding parameters.

NCMW: ▸ Docs.

This folder contains all documents related to how to work with the workflow. The documents are readable *via* https://manuelgloeckler.github.io/ncmw/as well.

NCMW: ▸ NCMW.

The main folder is organized into five parts: setup, analysis, community, utils, and visualization.

NCMW: ▸ NCMW ▸setup.

This is used to set the default bounds as specified in data/configs and the medium as specified in data/medium. It is also used to gap-fill the model with reactions or the medium with metabolites, such that all models obtain growth on the specified medium.

NCMW: ▸ NCMW ▸ analysis.

**FIGURE 2:**
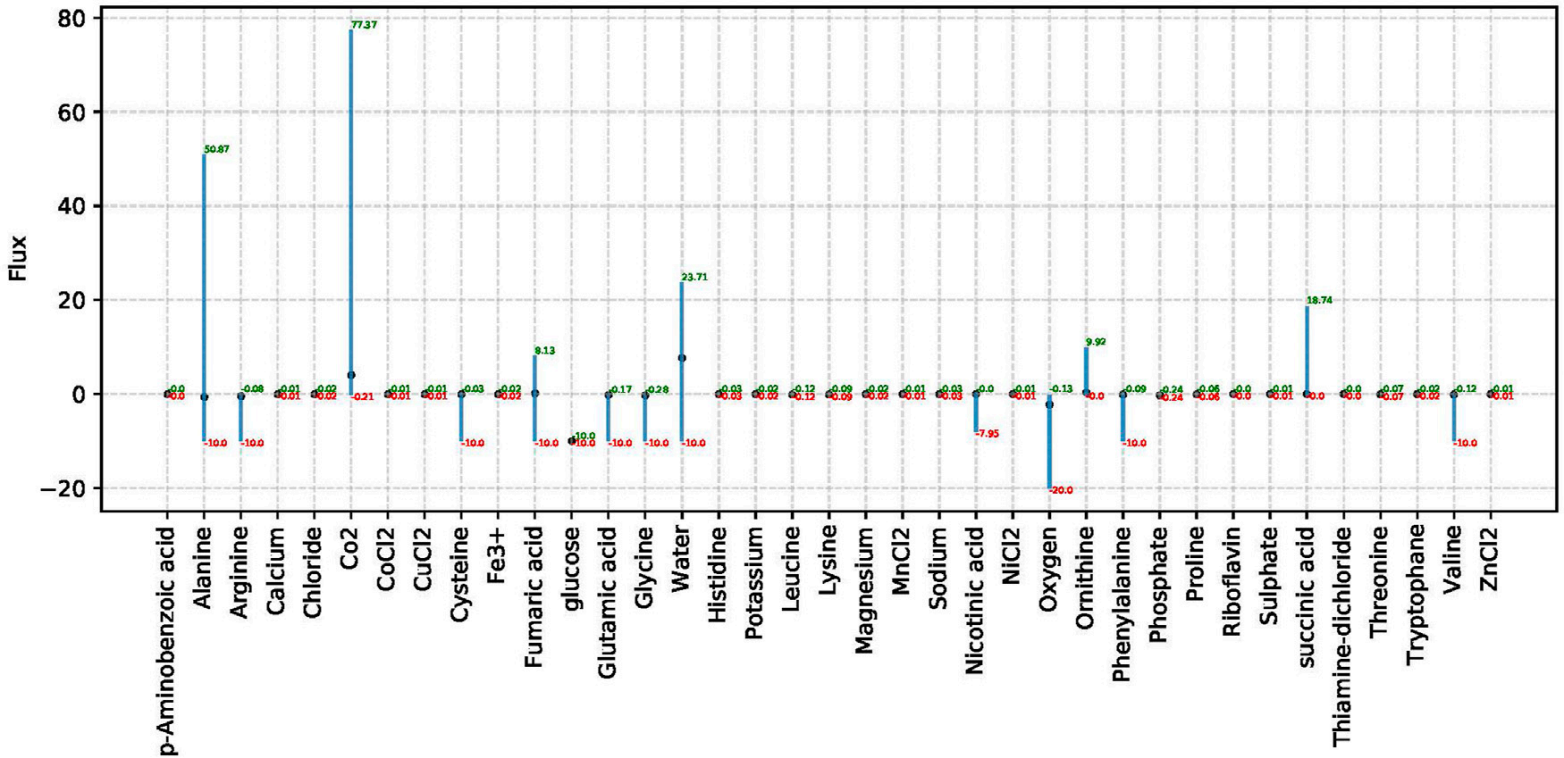
FVA results for non-zero exchange reactions in species *Dolosigranulum pigrum*. This analysis only takes non-zero exchange reactions into account based on the definition of the SNM3. The *x*-axis in this plot shows the associated metabolites to consider exchange reactions, and the *y*-axis shows the respective flux for the related exchange reaction.

**FIGURE 3:**
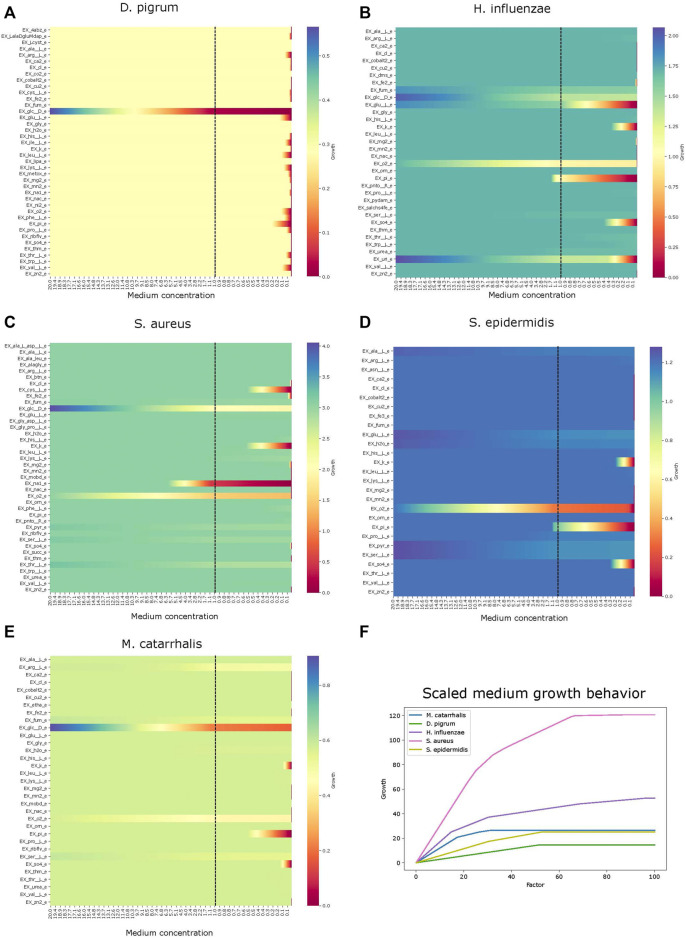
Effect of growth behavior on an increase of metabolites. Different models show how the growth scales are affected with abundance of metabolites. Panel **(A)** is species *Dolosigranulum pigrum*. Panel **(B)**, *Haemophilus influenzae*. Panel **(C)**, *Staphylococcus aureus*. Panel **(D)**, *Staphylococcus epidermidis*. Panel **(E)**, *Moraxella catarrhalis*. Panel **(F)**, growth comparison.

**FIGURE 4:**
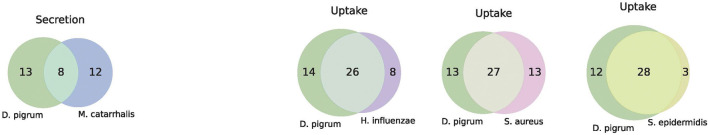
Major player within a community is what a model must uptake and what it can produce. Produced metabolites can benefit other community members, namely *Dolosigranulum pigrum*, *Haemophilus influenzae*, *Staphylococcus aureus*, *Staphylococcus epidermidis*, and *Moraxella catarrhalis*. However, too many shared uptakes can lead to competition within the community (resource overlap).

**FIGURE 5:**
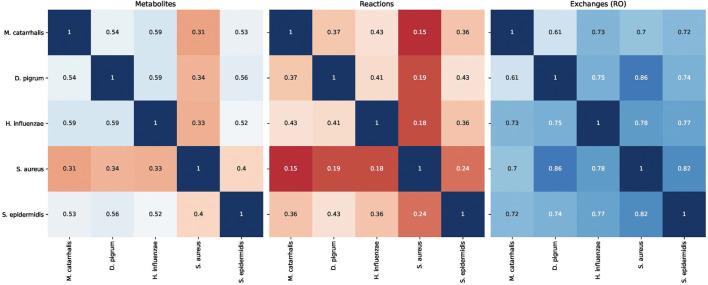
Similarity of different models of species *Dolosigranulum pigrum*, *Haemophilus influenzae*, *Staphylococcus aureus*, *Staphylococcus epidermidis*, and *Moraxella catarrhalis* based on the Jaccard distance.

Analysis performs flux variability analysis and visualizes results on all exchange reactions, as shown in Figure 2. This also shows the scaled growth behavior as shown in Figure 3. In addition, the uptake/secretion overlap between models is reanalyzed as shown in Figure 4. The similarity of models based on the number of shared metabolites/reactions as shown in Figure 5 is computed as well. COMPM, the medium in which all models are able to obtain their MBR, is computed.

NCMW: ▸ NCMW ▸ community.

**FIGURE 6:**
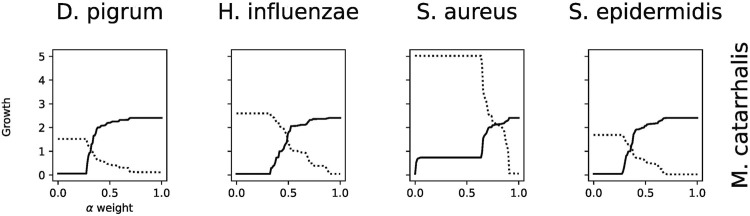
Growth of each model, given the weight *α*. This shows, e.g., at which weight the community is balanced between two species. For instance, the pair-wise interaction between species *Moraxella catarrhalis* and *Dolosigranulum pigrum*, *Haemophilus influenzae*, *Staphylococcus aureus*, and *Staphylococcus epidermidis* is plotted.

**FIGURE 7:**
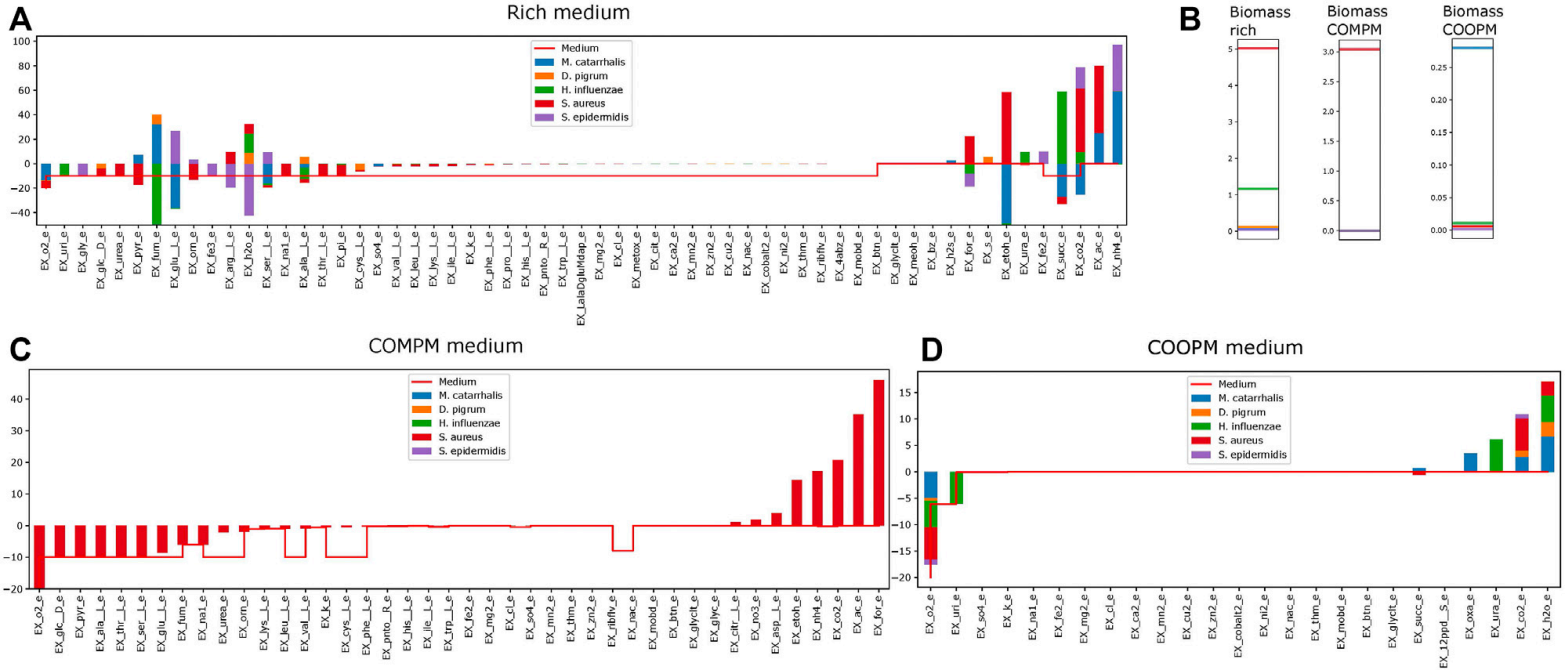
How much concentration of each metabolites are consumed or produced by each model when they are in the community for rich SNM3 [panel **(A)**], the COMPM [panel **(C)**], and the COOPM [panel **(D)**]. In panel **(B)**, we show the individual biomass rates obtained.

Community creates several kinds of community models, which all are based on Ebrahim et al., (2013). This also computes the COOPM, which is the smallest medium such that the community achieves 10% of the MBR, which induces cooperation. In addition, it visualizes the observed interactions between models, for instance, in Figure 6 and investigates the dependence of community weight and observed growth. This visualizes how the community looks like and visualizes the similarity between species in the community in consumption or production of metabolites as shown in Figure 7. In addition, it shows the growth of each species in the community.

NCMW: ▸ Output.

This folder contains all log files of each time run. A new folder that entitled the run date is automatically created and shows which command was applied while representing the log file.

NCMW: ▸ Results.

The results folder is automatically created with the default name defined. This is included in three main folders as setup, analysis, and community. Each contains the final results associated with the commands ncmw_setup, ncmw_analysis, and ncmw_community. Since the scripts automatically produce some results, NCMW, Data, *Hydra* helps the users to set up any block for each part of running scripts out of their interest.

NCMW: ▸ Tests.

Test folder includes test scripts related to commands ncmw_setup, ncmw_analysis, and ncmw_community.

### 3.2 Workflow

The NCMW scripts implement a series of tasks to carry out typical analysis procedures without any programming. We will discuss each component slightly based on the example of a five-member community consisting of *D. pigrum*, *S. aureus*, *M. catarrhalis*, *S. epidermidis*, and *H. influenzae* on the SNM3. To create the community of five members, the already existing GEMs of *D. pigrum* (Renz et al., 2021a) and *S. aureus* (Seif et al., 2019) were used. In addition, auto-generated GEMs of *S. epidermidis*, *H. influenzae*, and *M. catarrhalis* by CarveMe (Machado et al., 2018) version 1.2.2 in combination with DIAMOND (Buchfink et al., 2021) aligner version 0.9.26.127 were made. The information related to genome sequences of *S. epidermidis*, *H. influenzae*, and *M. catarrhalis* found in NCBI (Pruitt et al., 2005) is as follows:

1) *S. epidermidis*: The annotated genome sequence of *S. epidermidis* ATCC 12 228 (Zhang et al., 2003) included a NCBI Reference Sequence NC_004461.1 with the reference GCF_000007645.1 ^2^ and a total sequence length of 2,564,615.
2) *H. influenzae*: The annotated genome sequence of *H. influenzae* Rd KW 20 (Fleischmann et al., 1995; Tatusov et al., 1996) included a NCBI Reference Sequence NC_000907.1 with the reference GCF_000027305.1 ^3^ and a total sequence length of 1,830,138.
3) *M. catarrhalis*: The annotated genome sequence of *M. catarrhalis* BBH 18 (de Vries et al., 2013) included a NCBI Reference Sequence NC_014147.1 with the reference GCA_000092265.1 ^4^ and a total sequence length of 1,863,286.

#### 3.2.1 Setup of the Models

This part of the workflow considers two main parts: to check the quality of genome-scale metabolic models and the growth on the given SNM3. Most genome scale models are developed for the gut environment; thus, they often do not grow on a nasal medium.

##### 3.2.1.1 Quality Report of Genome-Scale Metabolic Models

The workflow starts with genome-scale metabolic models of the organism of interest, which have been already reconstructed, manually curated, and refined. The first step in the workflow is to check the quality of models. The models have been curated using the FastCC algorithm implemented in COBRApy (Vlassis et al., 2014). We further use Memote to create a quality report for each model (Lieven et al., 2020). A single improper model can strongly influence the community, i.e., a model that can produce much glucose out of nothing can enormously increase the biomass rates of all members to infinity [i.e., 1,000 mmol/(gDW⋅h)] within the community. Hence, this problematic behavior will become the dominant source of interaction and render the community useless for analysis. As a result, bad-quality models should be removed. The genome-scale metabolic models used here were all well curated and thus were kept for further analysis.

##### 3.2.1.2 The Growth Report of Models on SNM3

It has been reported whether the models are able to grow on SNM3, a medium mimicking the environment of the human nose. The exchange bounds of those metabolites defined for SNM3 can be set to a lower and upper bound. The default setting would be −10 mmol/(gDW⋅h) to 1,000 mmol/(gDW⋅h) for all metabolites defined in SNM3 except for oxygen and iron, which were set at −20 mmol/(gDW⋅h) to 1,000 mmol/(gDW⋅h) and −0.1 mmol/(gDW⋅h) to 1,000 mmol/(gDW⋅h), respectively. As explained in implementation of configs, the setting for bounds can be changed to the user’s interest. In addition, the default medium is SNM3, as the main focus of the workflow is the human nose community. However, implementation of the medium makes the possibility for defining any new medium based on the user’s interest.

The growth/no growth on SNM3 is reported in this step. In case of no growth, the workflow search for the missing metabolites that can be supplemented to the media allowed the organism’s growth on SNM3. This step is called the gap-filled step. The models with growth on SNM3 will be saved as an XML file. This makes their usage for further steps in the workflow smoother.

Alternatively, instead of supplementing the medium, we can add new reactions to the models to achieve growth. This alternative gap-filling strategy leaves the medium unchanged, yet one should typically check if the added reactions have supporting biological evidence.

Indeed, the workflow detected that *H. influenzae*, *D. pigrum*, and *M. catarrhalis* did not grow on the default SNM3. However, the gap-filling strategy succeeded. *M. catarrhalis* achieved a reasonable biomass rate of 1.2 mmol/(gDW⋅h) if we substitute EX_fe3_e in the medium. *H. influenzae* requires EX_uri_e to obtain a reasonable biomass rate of 1.7 mmol/(gDW⋅h). Our workflow shows that *D. pigrum* requires three new metabolites within the medium such as EX_LalaDgluMdap_e, EX_ile__L_e, and EX_metox_e. Renz et al. (2021a) identified l-isoleucine and l-methionine as well as 2,6-diaminoheptanedioate, which is required for peptidoglycan metabolism of *D. pigrum*. Our result consists of some similar chemical derivatives. The solution obtained by the workflow is not unique; both solutions are optimal (minimal number of metabolites), yet there may exist other combinations of metabolites to obtain growth.

#### 3.2.2 Analysis

This part majorly analyzes the models independently. This can give unique insights and understandings about the behavior within the community. The analysis focuses on exchange reactions, as internal reactions are less relevant for the community.

##### 3.2.2.1 Flux Analysis

For each model, the FBA and FVA are implemented to mimic the environment of the human nose. The FVA results in the range of values for which a specific model has a MBR. We show an example in Figure 2. In addition, the flux boundary for each species is computed by FVA and saved as tables and plots to show all non-zero exchange fluxes. FVA yields the upper and lower bounds for the fluxes through every reaction. With this, FVA can estimate the optimal solution when paired with the right combination of other fluxes as almost 100% of the maximum growth rate is achieved. The reactions sustaining low flux variability are presumably of higher priority to an organism. Consequently, FVA is a suitable method for identifying crucial reactions.

##### 3.2.2.2 Secretion Uptake

Using the FVA results, we can identify non-zero secreted and taken-up metabolites for each species, i.e., exchange reactions with strictly positive or negative flux. This step, as a starting point, helps the user determine how interactions can be. If we know what each model uptakes or secretes independently, we can draw certain conclusions about interactions within the community. If certain species have a similar set of uptake reactions, it may be reasonable to assume that they compete for common uptakes within the community. Alternatively, if one species secretes metabolites taken up by another, this may indicate a commensal interaction. To draw this conclusion, we must know if a particular metabolite, either added or removed, actually harms or benefits the growth rate of a species. This is shown in Figure 3, for each identified uptake reaction, we show the effect on the growth by changing the corresponding metabolite concentration within the medium. Figure 3 reveals that *D. pigrum* is highly glucose-dependent but requires many metabolites in small concentrations to obtain any growth. All other models admit more complex profiles, e.g., *S. aureus* also majorly depends on glucose, but growth can also be improved by, e.g., EX_pyr_e. Similarly, the growth of *H. influenzae* can be strongly improved by EX_glu__L_e, EX_fum_e, and EX_uri_e next to glucose.

##### 3.2.2.3 Growth

The default setting for exchange bounds is between −10 mmol/(gDW⋅h) and 1,000 mmol/(gDW⋅h) except for oxygen and iron. We scale this value by a factor *k* ∈ {1, … , 110} to see how the growth changes, and the results are reported as a plot. This compares the growth rate of all species to find their potential growth on an increasingly rich medium. The result is shown in Figure 3 **F**. Clearly, *S. aureus* has the largest growth potential and is thus expected to outcompete other species within the community.

##### 3.2.2.4 Similarity

These steps are ended by calculating the Jaccard index between species to find the similarity between reactions and metabolites of all species in one glance. Interestingly, all models are somewhat similar to each other, except *S. aureus*. This may explain the difference within the potential growths. Additionally, it shows the resource overlap, i.e., the Jaccard similarity of exchange reactions, e.g., *D. pigrum* and *S. aureus* have high similarity. Thus, we may expect that they compete for similar resources.

##### 3.2.2.5 Medium

To design a community, we need to define which metabolites should be shared in the community since the interaction analysis would be feasible for experimental validation if not taking all the significant number of union metabolites into account. To this end, the COMPM defined in the *Materials and Methods* part is computed as a minimum of min FVA fluxes for those exchange reactions that are common and only take the corresponding min FVA flux for uncommon ones and are saved as a JavaScript Object Notation (JSON) file. This medium is a subset of the default medium, based on our setting, which is SNM3.

#### 3.2.3 Community

Last but not least, we create a community model to analyze the interaction between species as a pooled or compartmentalized community with the shuttle reaction. Here, we focus on the compartmentalized community as it allows detailed investigation into metabolite exchange.

##### 3.2.3.1 Computation of the Maximal Biomass Rate of the Communities

The computation of the MBR for the community is possible by setting which approach for construction of the community is used and which defined medium is applied. Additionally, due to different growth between species, the weights used to compute objective functions in the community can be set manually. The weights can represent the (relative) abundances of each species within the community, yet this assumes that the biomass rate of each model is well calibrated concerning each other. Alternatively, we can see them as free parameters which can be set to achieve a realistic community behavior. Analyzing the community with uniform weights can be challenging if a single species can achieve a comparably high biomass rate. Thus, the community solution will only allow this community member to have growth. Hence, the workflow implements several methods to counteract this problem. First, we can set “fair” weights, which are inversely proportional to the individual biomass rates. Therefore, the growth within the community is not only majorly determined by individual biomass rates but also how good or bad the interaction with other community members is. Alternatively, we can change the community objective to either ensure that community members must reach a certain percentage of community growth or use cooperative trade-off (Diener et al., 2020).

##### 3.2.3.2 Community Summary

The community summary report contains all exchanges between community members or the medium. To show how the community summary works, for instance, a community summary of five species is visualized in Figure 7, while uniform weights are applied. On the rich SNM3, the model predicts that *S. aureus* and *H. influenzae* admit significant growth rates, whereas all other community members are close to zero. As glucose majorly affects the growth of *S. aureus*, it indeed uptakes the majority of glucose within the medium as visualized in Figure 7. Glucose is only shared with *D. pigrum*, the second major glucose-dependent species. However, this glucose deficit seems to be compensated by *S. aureus* through the uptake of EX_pyr_e (which we identified previously as beneficial for growth (Mostolizadeh et al., 2022) and experimentally observed that pyruvate was consumed instead of glucose to produce lactate for balancing the reducing equivalents (NAD+) (Carvalho et al., 2017)). This excess amount is not provided by the medium but by *M. catarrhalis*. On the other hand, *M. catarrhalis* and *D. pigrum* provide EX_fum_e, which is the major contributor to the growth of *H. influenzae*, which then provides outstanding amounts of EX_succ_e for *M. catarrhalis* and *S. aureus*. This serves as one example of a complex commensal interaction cycle that can be revealed by metabolic community modeling.

The COMPM induces more competition for resources as indeed the standard community objective only supports the growth of *S. aureus*, which outcompetes all other species due to its high individual growth rate (see Figure 7).

The COOPM represents the smallest medium in which all community members can (slightly) grow. Notice that the medium does not contain any glucose. *M. catarrhalis* is now the dominant community member as it can achieve comparably high biomass rates even without any glucose.

##### 3.2.3.3 Prediction of Pair-Wise Interaction

Freilich et al. (Freilich et al., 2011) designed two different formulas as a potential competition score (PCMS) and a potential cooperation score (PCPS), to quantify the level of competition and cooperation predicted among the species.

$${\text{PCMS}}_{AB}=1-\frac{V_{BM,\text{COMPM},AB}-max\left(V_{BM,\text{COMPM},A},V_{BM,\text{COMPM},B}\right)}{V_{BM,\text{COMPM},A}+V_{BM,\text{COMPM},B}-max\left(V_{BM,\text{COMPM},A},V_{BM,\text{COMPM},B}\right)};$$ (2)

$${\text{PCPS}}_{AB}=1-\frac{V_{BM,\text{COOPM},A}+V_{BM,\text{COOPM},B}}{V_{BM,\text{COOPM},AB}},$$ (3)

where *V*_*BM*,*x*,*y*_ represents the MBR of species *y* in the community *x*. If the PCMS value equals 0, then this denotes no competition, while one indicates maximal competition. In addition, the negative PCMS values and positive PCPS values stand for cooperation, while negative PCPS values indicate competition. Therefore, the workflow determines the level of competition and cooperation between species by comparing their individual and combined biomass rates across simulated communities in the COMPM and COOPM.

**FIGURE 8:**
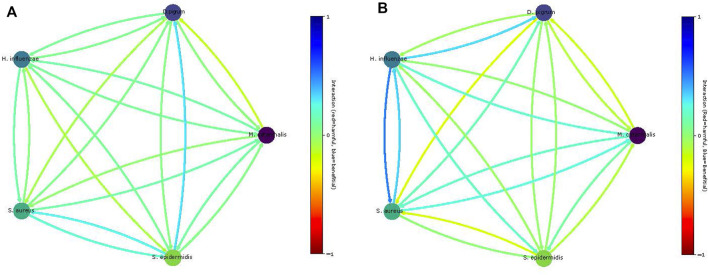
Predicted community interactions within different media. Panel **(A)** shows interaction on the rich SNM3. Panel **(B)** shows the interaction on the COMPM under cooperative trade-off.

These two equations Eq. 2 and Eq. 3 are applied to computationally predict the pair-wise interaction between species and compare our results with experimental ones. In Figure 8, we show the computationally obtained values. Notice that on the COMPM, only *S. aureus* achieved growth. Thus, no interactions can be determined. Nevertheless, by using cooperative trade-off, we obtain a less sparse solution as shown in Figure 8. Clearly, the competition among species increased. Here, the negative interaction of *D. pigrum* to *S. aureus* becomes more apparent. However, the interaction of *S. aureus* to *D. pigrum* is slightly commensal (Mostolizadeh et al., 2022). As shown in Mostolizadeh et al., (2022), *S. aureus* can provide relevant metabolites to *D. pigrum*, yet *D. pigrum* is a major competitor for glucose.

##### 3.2.3.4 Ability of the Model to Provide Metabolites for its Members’ Growth

Genome-scale modeling for each species creates a situation that rarely occurs in nature because it considers each species isolated. In real-life environments, species usually thrive in complex communities in which the growth of a single species depends on the interactions with other species in the population. Our workflow makes this analysis possible to find out how species can influence each other’s growth. To this end, first, essential metabolites necessary for the growth of each single species in SNM3 are found. Second, additional constraints are added to our community to allow the exchange of those metabolites between species if they are among the exchange reactions of other species. Those required metabolites that are not found among the exchange reactions of other species are manually added to the community since there are no species to provide it.

### 3.3 Availability and Requirements

Project name: NCMW.

Project documentary page: https://manuelgloeckler.github.io/ncmw/

Operating systems: MacOS, Windows, Linux.

Programming language: Python (version >3.7).

License: MIT License.

## 4 Discussion

The open-source package NCMW simulates the microbe–microbe interactions to show how important their roles in shaping the composition of human nasal microbiota are. However, the cultural capability of strains in microbial communities in the laboratory remains limited. Since this community research in the human nose provides explicit, testable hypotheses and potential targets for experimental verification, it provides a basis to examine the therapeutic potential of individual species as a typical nasal probiotic and possible novel discoveries.

We applied a combination of different optimization approaches to analyze the nasal microbial community to determine what a well-matched theoretical and experimental approach would be. Therefore, two stages are taken into account. One can be the optimization of the objective functions, and the other is the process of deciding what kind of trade-offs are appropriate from the decision-maker’s perspective. These two stages classify the techniques into three techniques: The decision-maker, the designer, is the set of optimal compromise solutions that an effective and complete search procedure must identify to carry out the best choice (Chiandussi et al., 2012). Therefore, these techniques are discussed by analyzing some of their advantages and disadvantages.

1) *Priori* technique: It takes decisions before searching and includes those approaches that assume that either a specific desired achievable goal or a certain preordering of the objectives can be performed by the decision-maker prior to the search.
2) *Posteriori* technique: It searches before making decisions and does not require prior preference information from the decision-maker.
3) Progressive technique: It integrates search and decision-making.

The big advantage of the package is its flexibility in creating the different types of communities to convey the way for analysis. In addition, it builds a community in the *de facto* standard format SBML Level 3 Version 1 (Hucka et al., 2018; Keating et al., 2020; Renz et al., 2021b) with flux balance constraints (FBC) extension version 2 (Olivier and Bergmann, 2018), making any further analysis on the community smoother. Although the workflow was reconstructed for the nasal microbiome community, it is also possible to apply it for any model in other communities. The package automatically creates media defined in the input model and saves it as a JSON file to proceed with the research. As a disadvantage, when more models are added to the community, more running time is expected. However, to shorten the running time, one can use high-quality models to ignore using quality checks of models. In addition, defining a small medium that included only a few metabolites makes understanding a complex community smoother but far from a realistic way.

## Data Availability

The original contributions presented in the study are included in the article/Supplementary Material; further inquiries can be directed to the corresponding author.

## Footnotes

1
https://cobrapy.readthedocs.io/en/devel/autoapi/cobra/flux_analysis/fastcc/index.html.

2
https://www.ncbi.nlm.nih.gov/assembly/GCF_000007645.1.

3
https://www.ncbi.nlm.nih.gov/assembly/GCF_000027305.1.

4
https://www.ncbi.nlm.nih.gov/assembly/GCA_000092265.1.
